# Effect of Na_2_O on the Low-Temperature Densification, Crystallization Behavior, and Dielectric Properties of Perlite Tailings-Derived α-Cordierite Glass-Ceramics

**DOI:** 10.3390/ma19071348

**Published:** 2026-03-28

**Authors:** Saibo Wang, Yongsheng Yu, Yunxiao Zhao, Pengzhen Wang, Jinghan Wang, Zhaoli Yan, Qiangshan Jing

**Affiliations:** Henan Province Key Laboratory of Utilization of Non-Metallic Mineral in the South of Henan, Xinyang Normal University, Xinyang 464000, China

**Keywords:** LTCC, cordierite glass-ceramics, perlite tailings, Na_2_O, crystallization behavior

## Abstract

To facilitate the development of low-cost LTCC substrate materials and the high-value utilization of industrial tailings, α-cordierite glass-ceramics with varying Na_2_O additions were prepared from perlite tailings as the main raw material via the melt-quenching method followed by sintering-induced crystallization. The synergistic effects of sintering temperature and Na_2_O addition on the parent glass structure, crystallization behavior, and properties were systematically investigated. The results demonstrated that the addition of Na_2_O effectively depolymerized the degree of network polymerization of the parent glass, altered the crystallization pathway of cordierite crystal, and promoted the densification of glass-ceramics at lower sintering temperature. The calculations of crystallization kinetics revealed that the crystallization process of α-cordierite was mainly dominated by three-dimensional bulk growth, and its nucleation mechanism changed from “site saturation” to “continuous nucleation” with the increase of Na_2_O addition. The α-cordierite glass-ceramics sintered at 850 °C with 0.6 wt.% Na_2_O addition exhibited the optimal comprehensive properties, including low dielectric constant (5.82 @ 10 MHz) and dielectric loss (1.80 × 10^−2^ @ 10 MHz), high flexural strength (147.3 MPa), a Vickers hardness (9.01 GPa), and suitable coefficient of thermal expansion (2.96 × 10^−6^ K^−1^, close to Si). The glass-ceramics are expected to be an ideal candidate for low-cost LTCC substrate materials.

## 1. Introduction

In recent years, driven by the rapid advancement of emerging technologies such as virtual reality, the Internet of Things (IoT), and artificial intelligence, Low Temperature Co-fired Ceramics (LTCC) technology has garnered significant attention owing to its superior high-frequency dielectric properties and advantages in integration [1,2,3,4]. Among various LTCC substrate materials, cordierite glass-ceramics are regarded as one of the most promising candidates, owing to their low dielectric constant (ε_r_ ≈ 5), low dielectric loss (tanδ ≈ 10^−3^), and a coefficient of thermal expansion that matches well with chip materials [5,6,7,8,9]. However, the fabrication of traditional cordierite glass-ceramics typically relies on high-purity chemical raw materials. The resulting high cost hinders their penetration into large-scale markets, such as consumer electronics. Perlite, a vitreous volcanic rock rich in aluminosilicates, is primarily composed of 70–75 wt.% SiO_2_, 12–15 wt.% Al_2_O_3_, and 6–9 wt.% alkali metal oxides, a composition that aligns well with the requirements for cordierite synthesis [10]. The utilization of perlite tailings as a substitute for pure chemical raw materials not only significantly reduces costs but also optimizes the sintering process due to their inherent glassy nature, offering substantial scientific value and economic benefits.

However, the fabrication of cordierite glass-ceramics derived from perlite tailings still faces significant challenges, including the high viscosity of the parent glass at elevated temperatures, a narrow sintering window, and the difficulty in achieving full densification at lower temperatures. Existing studies have shown that the incorporation of alkali metal oxides, such as K_2_O [11], Li_2_O [12] and Na_2_O [12], is one of the effective strategies to address these issues. As typical network modifiers, alkali metal oxides can depolymerize the glass network structure and reduce the degree of network polymerization [13,14], thereby significantly lowering the melt viscosity and promoting the low-temperature densification sintering of glass-ceramics.

Several studies have investigated the effects of alkali metal oxides on the structure and crystallization behavior of MAS-based glasses and glass-ceramics. Wang et al. [15] systematically examined the influence of different alkali metal oxides (Na_2_O, Li_2_O and K_2_O) on the high-temperature viscosity and crystallization behavior of MgO-Al_2_O_3_-SiO_2_ glass using TiO_2_ + ZrO_2_ as nucleating agents. The results showed that the introduction of alkali metal oxides could effectively reduce the viscosity of the glass melt and significantly affect the transparency of the resulting glass-ceramics by altering the crystallization pathway and crystalline phase constitution. Guo et al. [16] reported that, in Na_2_O-MgO-Al_2_O_3_-SiO_2_ glass, increasing the Na_2_O content decreased the degree of glass network polymerization, thereby lowering the elastic modulus and increasing the coefficient of thermal expansion; however, its effect on crystallization behavior was not systematically investigated. Song et al. [11] used the natural mineral potash feldspar as a raw material and investigated the effects of different chemical compositions on the crystallization kinetics of α-cordierite glass-ceramics by varying the amount of potash feldspar added and the stoichiometric ratio. The results showed that an appropriate amount of K^+^ could effectively reduce the crystallization activation energy of α-cordierite, confirming that the alkali metal components in natural mineral raw materials play an important role in promoting low-temperature crystallization. Veselov et al. [17] pointed out that, in ZnO-MgO-Al_2_O_3_-SiO_2_ glass, an increase in Na_2_O content was beneficial for enhancing the efficiency of Na^+^-K^+^ ion exchange, increasing the surface compressive stress and hardness, and lowering both the glass transition temperature and the initial hardness. He et al. [18] systematically investigated the effects of equimolar substitution of Na_2_O by Li_2_O on the glass-forming ability of NMAS glass, as well as on the microstructure, mechanical properties, crystallization behavior, and ionic conductivity of the corresponding glass-ceramics. The results indicated that variations in the proportion of alkali metal oxides induced a pronounced mixed-alkali effect, leading to nonlinear changes in the glass transition temperature, mechanical properties, and ionic conductivity of the parent glass. Meanwhile, by modifying the degree of glass network polymerization, crystallization pathway, and phase composition, the alkali metal oxide ratio markedly regulated the crystallinity and ion transport behavior of the glass-ceramics. A comparative study by Kang et al. [12] further demonstrated that Li^+^, with its high field strength, could significantly reduce the crystallization activation energy of μ-cordierite and promote its precipitation at lower temperatures. In contrast, replacing Na^+^ with K^+^, which has a larger ionic radius, suppressed the diffusion process, increased the crystallization activation energy, and hindered the crystallization of cordierite.

These studies demonstrate that alkali metal oxides can markedly influence the glass structure, melt viscosity, crystallization kinetics, phase composition, and related properties of MAS-based systems. However, existing research has mainly focused on conventional MAS model systems or on specific topics such as single-alkali substitution, ion-exchange strengthening, and transparent glass-ceramics. In contrast, the effects of Na_2_O on the glass structure, phase transformation pathway, low-temperature densification, and the mechanical and dielectric properties of perlite tailings-derived cordierite glass-ceramics have not yet been systematically investigated.

In light of this, the present work focuses on the preparation of cordierite glass-ceramics with varying additions of Na_2_O, utilizing perlite tailings as a partial raw material. The effects of sintering temperature and Na_2_O addition on the glass network structure, crystallization behavior, microstructure, and sintering properties were systematically investigated. Furthermore, by incorporating non-isothermal crystallization kinetics analysis, the specific roles of Na_2_O in modifying the activation energy for crystallization, as well as the nucleation and crystal growth mechanisms, were elucidated. These findings provide valuable references for the high-value-added utilization of perlite tailings and the design of low-cost, high-performance LTCC substrate materials.

## 2. Experimental Procedures

### 2.1. Preparation of Glasses and Glass-Ceramics

The starting materials employed in this work were MgO, Al_2_O_3_, and SiO_2_ (99.7%, China National Pharmaceutical Group Chemical Reagent Co., Ltd., Shanghai, China), together with Na_2_CO_3_ (99.0%, Shanghai Aladdin Biochemical Technology Co., Ltd., Shanghai, China) and Shangtianti perlite tailings obtained from Xinyang, China. The detailed chemical composition of the perlite tailings is listed in Table 1.

In this study, parent glasses were first prepared by melt-quenching, and the cordierite glass-ceramics were subsequently obtained by sintering-induced crystallization. In the batch design, the total batch mass was normalized to 100 wt.%. The content of perlite tailings was fixed at 45 wt.%, while additional Na_2_O was introduced at x wt.% (x = 0.0, 0.3, 0.6 and 1.1). The remaining components were supplemented by added MgO, Al_2_O_3_ and SiO_2_. By taking into account the chemical composition of the tailings and adjusting the proportions of the added oxides, the overall molar ratio of MgO:Al_2_O_3_:SiO_2_ in the system was maintained at 2.6:2:5. Here, x denotes the additional Na_2_O introduced beyond that inherently contained in the perlite tailings. Accordingly, the total Na_2_O contents in the parent glasses were 1.24, 1.54, 1.84 and 2.34 wt.%, respectively. The final chemical compositions of all samples are listed in Table 2. The selected Na_2_O levels were determined based on preliminary compositional consideration and exploratory experiments. Specifically, 0 wt.% was used as the control composition, 0.3 and 0.6 wt.% were chosen to evaluate the progressive modification effect of Na_2_O within the low-addition regime, and 1.1 wt.% was intentionally selected as an upper-bound composition to probe the onset of excessive modification, including secondary-phase formation and reduced phase stability. The batched raw materials were first blended uniformly by wet ball milling. After drying, the mixed powders were transferred into a corundum crucible and heated from ambient temperature to 1550 °C at a rate of 10 °C/min, followed by a 6 h dwell at 1550 °C. The resulting homogeneous melt was subsequently quenched in water to form glass blocks. These glass pieces were crushed and further ground by wet ball milling to prepare the parent glass powder. An appropriate quantity of the obtained powder was then combined with a 5 wt.% PVA solution to generate granulated material. The granules were compacted into cylindrical green compacts under a uniaxial pressure of 20 MPa. Afterward, the green specimens were heated in air from room temperature to 825–875 °C at a fixed heating rate of 10 °C/min and maintained for 6 h for sintering. Finally, the sintered bodies were cooled down to room temperature inside the furnace, yielding the final glass-ceramic samples.

### 2.2. Analytical Methods

Differential scanning calorimetry (DSC, STA 449 F5, NETZSCH-Gerätebau GmbH, Selb, Germany) was carried out in air using an empty alumina crucible as the reference. The parent glass powder was heated from room temperature to 1200 °C at a rate of 10 °C/min to determine the glass transition and crystallization temperatures. To investigate the crystallization kinetics of the glass-ceramics, DSC measurements were further conducted at heating rates of 5, 10, 20, and 30 °C/min. The phase composition of the parent glass and glass-ceramics was analyzed by X-ray diffraction (XRD, MiniFlex 600, Rigaku Corporation, Akishima, Tokyo, Japan) with Cu Kα radiation over a 2θ range of 5–80°. A scanning rate of 10°/min was used for phase identification, while the patterns for Rietveld refinement were collected at 2°/min. The microstructure and surface morphology of the fracture surfaces and hydrofluoric acid-etched surfaces were examined by field-emission scanning electron microscopy (FESEM, S-4800, Hitachi High-Tech Corporation, Tokyo, Japan). The thermal expansion coefficient was measured using a dilatometer (Netzsch DIL-402C, NETZSCH-Gerätebau GmbH, Selb, Germany) under a nitrogen atmosphere from 30 to 600 °C at a heating rate of 5 °C/min. Flexural strength was determined by the three-point bending method using an electric bending tester (KZJ-300N, Shenyang Tianping Instrument Co., Ltd., Shenyang, China), with a span of 20 mm and a loading rate of 4 N/s. The bulk density was determined by the Archimedes method. Fourier transform infrared spectroscopy (FTIR, Nicolet iS50, Thermo Fisher Scientific, Waltham, MA, USA) was employed to record the infrared spectra of the parent glass and glass-ceramics in the range of 400–1400 cm^−1^. The dielectric properties, including dielectric constant and dielectric loss, were measured using a precision impedance analyzer (E4990A, Keysight Technologies, Santa Rosa, CA, USA) in the frequency range of 20 Hz–10 MHz. The O 1s spectrum of the parent glass was characterized by X-ray photoelectron spectroscopy (XPS, K-Alpha+, Thermo Fisher Scientific, East Grinstead, West Sussex, UK).

## 3. Results and Discussion

### 3.1. DSC Analysis

**Figure 1:**
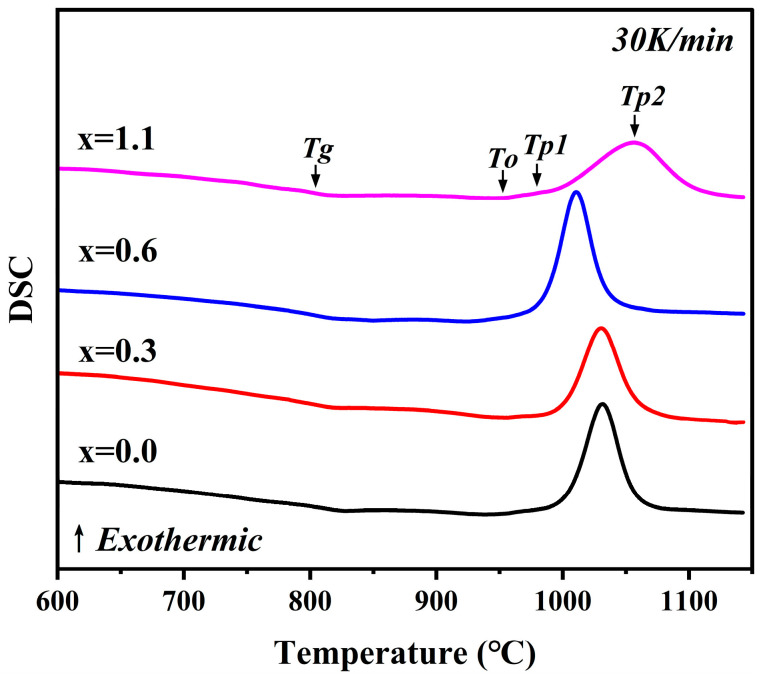
DSC curves of parent glasses with varying Na_2_O additions at a heating rate of 30 K/min.

Figure 1 illustrates the DSC curves of the MAS parent glasses with varying Na_2_O additions, measured at a heating rate of 30 °C/min. It can be observed that, within the temperature range of 750 °C to 1100 °C, all glass samples exhibit similar thermal response behaviors, characterized by an endothermic glass transition region followed by two exothermic crystallization peaks. Based on the subsequent XRD analysis of the glass-ceramic samples, the first exothermic peak corresponds to the crystallization of the metastable μ-cordierite phase, while the second exothermic peak corresponds to the crystallization of α-cordierite. Table 3 systematically summarizes the key thermodynamic parameters of the parent glasses, which are determined by their chemical compositions and structural characteristics.

As indicated in Figure 1 and Table 3, with the Na_2_O addition gradually increasing from 0 wt.% to 0.6 wt.%, the glass transition temperature (*T*_g_), the onset crystallization temperature (*T*_o_), the crystallization peak temperature of μ-cordierite (*T*_p1_), and the crystallization peak temperature of α-cordierite (*T*_p2_) all shift toward lower temperatures. However, when the Na_2_O addition is further increased to 1.1 wt.%, while *T*_g_ continues to shift toward lower temperatures, both *T*_o_, *T*_p1_ and *T*_p2_ shift toward higher temperatures. This indicates that the addition of Na_2_O can promote the precipitation of cordierite crystals at lower temperatures; however, this promotional effect does not follow a monotonic relationship. Excessive Na_2_O, conversely, inhibits the formation of cordierite crystals, even though the glass transition process of the parent glass is completed at a lower temperature.

Na_2_O is a typical network modifier in aluminosilicate glass systems. Upon incorporation into the glass structure, it disrupts part of the bridging oxygen bonds (BO), such as Si-O-Si and Al-O-Si linkages, and promotes the formation of additional non-bridging oxygens, thereby reducing the degree of polymerization and connectivity of the glass network [19,20]. As network depolymerization intensifies, the rigidity of the structural framework weakens and the average bond strength decreases, which lowers the kinetic barrier that must be overcome for cooperative structural relaxation during the glass transition [21]. Consequently, the base glass can complete the glass transition at a lower temperature, macroscopically manifested as a shift of *T*_g_ toward lower temperatures.

The crystallization behavior of the parent glass is co-regulated by multiple factors, including glass composition, network structure, and ion diffusion kinetics [19,20]. As a typical glass network modifier, the introduction of Na_2_O weakens and partially depolymerizes the original three-dimensional network structure of the parent glass. This leads to a decrease in the content of BO and an increase in the content of non-bridging oxygens (NBO), thereby reducing the overall degree of polymerization of the glass network [21]. These variations in glass structure are verified by the subsequent semi-quantitative analysis of the relative contents of BO and NBO via FTIR and XPS. Furthermore, due to the low cationic field strength of Na^+^, its ability to polarize oxygen ions is limited; the resulting weak ionic bonds cannot maintain high skeletal rigidity, leading to a decline in structural stability. This structural weakening is inferred to reduce the melt viscosity and the kinetic barrier for particle migration [22,23,24]. Consequently, the diffusion and rearrangement of nucleating components such as Mg^2+^ and Al^3+^ are accelerated, facilitating nucleation and crystal growth, which is macroscopically manifested as a decrease in crystallization temperature. However, for the sample with x = 1.1, the rebound in crystallization temperature may be attributed to the thermodynamic inhibition effect overriding the kinetic promotion effect. According to classical nucleation theory, the introduction of excessive Na^+^ lowers the free energy of the melt, thereby reducing the free energy difference relative to the cordierite crystal phase [25,26,27]. This, in turn, diminishes the thermodynamic driving force for the transformation from the liquid phase to the crystalline phase. In addition, the presence of a large amount of Na^+^ may also reduce the compatibility between the local structure of the glass melt and the cordierite crystal structure, as well as create diffusion hindrance to crystal growth [25,26,27]. This increases the crystal-liquid interfacial energy and the nucleation barrier. Simultaneously, excessive Na^+^ promotes the formation of Na-containing impurity phases. The competitive relationship between these crystal phases also inhibits the formation of cordierite crystals. This hypothesis is supported by the subsequent detection of the nepheline phase in the XRD analysis of the glass-ceramic samples. Consequently, this is macroscopically manifested as an increase in the crystallization temperature of the glass.

### 3.2. Non-Isothermal Crystallization Kinetics

**Figure 2:**
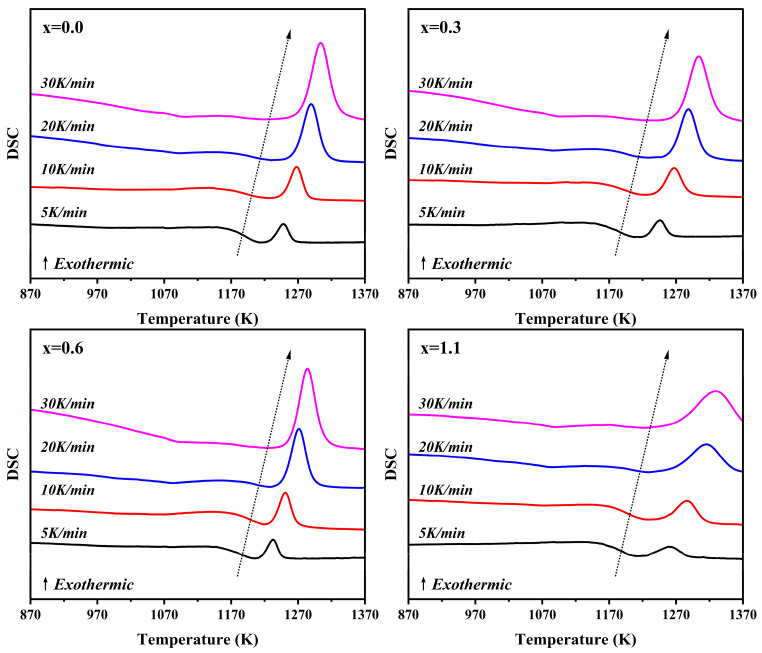
DSC curves of parent glasses with varying Na_2_O additions at different heating rates.

Figure 2 presents the DSC curves of parent glasses with varying Na_2_O additions, measured at different heating rates (*β* = 5, 10, 20 and 30 °C/min). From Figure 2, it can be observed that for a given sample, *T*_p_ shifts toward higher temperatures as *β* increases. This is a typical phenomenon in non-isothermal crystallization kinetics. Under linear heating conditions, the variation of the crystallization conversion fraction, α, can be expressed by Equation (1) [28,29]. At the same temperature *T*, a larger *β* results in a smaller d*α*/d*T*. During the heating process, the system is unable to complete sufficient nucleation and early-stage growth within the lower temperature range in time, leading to a reduction in the actual conversion fraction at any given temperature.

(1) $$\frac{d\alpha }{dT}=(\frac{1}{\beta })k(T)f(\alpha )$$

where *α* is the fraction of crystallization (conversion percentage), *β* is the heating rate, and k(*T*) is the reaction rate constant.

The crystallization activation energy (*E*) refers to the energy barrier that structural units must overcome to rearrange during the transition from the glassy state to the crystalline state. It serves as a critical indicator for evaluating the crystallization capability of the parent glass. To deeply investigate the dynamic modulation effect of Na_2_O on the crystallization behavior of cordierite glass-ceramics, this study calculated *E* using the Kissinger [30] and Ozawa [31] methods based on multi-heating-rate DSC data (*β* = 5, 10, 20 and 30 °C/min). Furthermore, the Avrami exponent (*n*) was calculated by combining these results with the Matusita–Sakka model [32] to characterize the non-isothermal nucleation and crystallization mechanisms. The Kissinger and Ozawa equations are given as follows:

(2) $$\mathrm{Kissinger}\;\mathrm{equation}:\ln(\frac{\beta }{{T_{p}}^{2}})=-\frac{E}{RT_{p}}+\mathrm{constant}$$

(3) $$\mathrm{Ozawa}\;\mathrm{equation}:\ln\beta =-\frac{E}{RT_{p}}+\mathrm{constant}$$

where *T*_p_ is the peak crystallization temperature, *β* is the heating rate, and *R* is the ideal gas constant.

**Figure 3:**
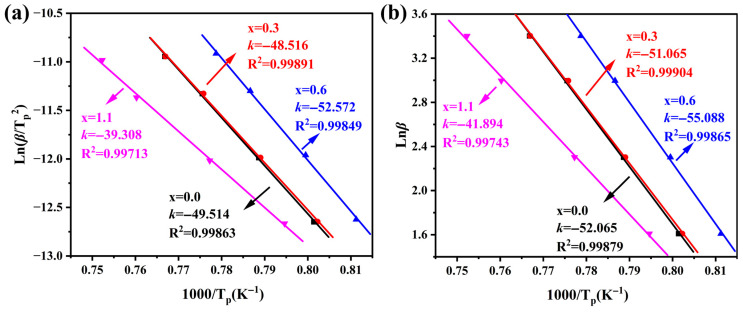
Kissinger fitting plots of ln(*β*/*T*_p_^2^) versus 1000/*T*_p_. (**a**), Ozawa fitting plots of ln*β* versus 1000/*T*_p_ (**b**), for parent glasses with different additions of Na_2_O.

The Kissinger and Ozawa methods derive *E* through the linear fitting of ln(*β*/*T*_p_^2^) versus 1000/*T*_p_ and ln*β* versus 1000/*T*_p_, respectively. The corresponding linear fitting results are illustrated in Figure 3, and the specific calculated *E* values for α-cordierite are listed in Table 4. As the Na_2_O addition increases, *E* of α-cordierite generally exhibits a downward trend. This indicates that the addition of Na_2_O lowers the crystallization energy barrier for α-cordierite, facilitating its precipitation at lower temperatures. It is worth noting that the sample with x = 0.6 exhibits a relatively larger *E*, which may be related to changes in the crystallization behavior of the metastable intermediate phase, μ-cordierite, at this composition. If the formation of the intermediate μ-cordierite phase is inhibited, the number of heterogeneous nucleation sites it can provide decreases, thereby resulting in a higher observed crystallization energy barrier for α-cordierite. It is noteworthy that the activation energy values obtained by the Kissinger and Ozawa methods are relatively close, and both exhibit a consistent variation trend with increasing Na_2_O addition, indicating that the non-isothermal crystallization kinetics analysis in this study possesses good internal consistency and reliability.

**Figure 4:**
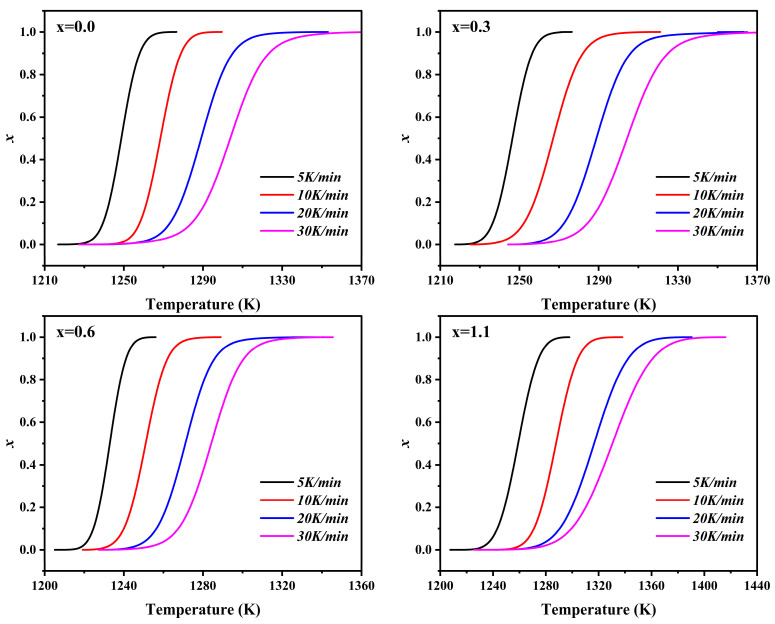
The crystalline volume fraction *x* of parent glasses with different additions of Na_2_O as a function of temperature (*T*) at different heating rates.

The crystal volume fraction *x* can be expressed as [33,34]:

(4) $$x=\frac{\Delta H_{T}}{\Delta H}=\frac{\int \begin{array}{l} T\\ T_{o} \end{array}\frac{dH}{dT}dT^{\prime }}{\int \begin{array}{l} T_{e}\\ T_{o} \end{array}\frac{dH}{dT}dT^{\prime \prime }}$$

where *T*_o_ and *T*_e_ are the onset and end temperatures of crystallization, respectively, ∆*H_T_* is the crystallization enthalpy in the temperature range from *T*_o_ to *T*, and ∆*H* is the total enthalpy of crystallization. Figure 4 shows the crystallized volume fraction (*x*) versus temperature (*T*) curves for parent glasses with varying Na_2_O additions at different heating rates. The slope of the *x*–*T* curves in the figure reflects the crystallization rate of the parent glass. The *x*–*T* curves for all samples exhibit a typical sigmoidal shape, and the crystallization process can generally be summarized into three stages. In the initial stage of crystallization (*x* < 0.2), the high viscosity of the glass melt restricts atomic diffusion and rearrangement, resulting in a low crystallization rate. As the temperature rises, both the atomic diffusion rate and nucleation efficiency increase rapidly, leading to a sharp increase in the crystallization rate; this marks the entry into the rapid crystallization stage (*x* = 0.2–0.8). In the final stage of crystallization (*x* = 0.8–1.0), the impingement effect of adjacent crystal grains and the substantial consumption of crystallizable components extend the diffusion path, causing the crystallization rate to gradually decay and approach zero [35].

*N* serves as a pivotal parameter in elucidating crystallization mechanisms, as its numerical value quantitatively characterizes both the dimensionality of crystal growth and the temporal dependence of the nucleation rate. The classical Matusita–Sakka equation is used to calculate the n of the samples [32]:

(5) $$\ln[-\ln(1-x)]=-n\ln\beta -1.052\frac{mE}{RT}+\mathrm{constant}$$

where *x* is the crystallization volume fraction, *n* is the Avrami index, *β* is the heating rate, *E* is the crystallization activation energy, and *R* is the ideal gas constant. Then, ln[−ln(1 − *x*)] is plotted against in *β*, and the derived data points are linearly fitted through the least squares method, leading to the slope of the line, i.e., the −*n* value.

**Figure 5:**
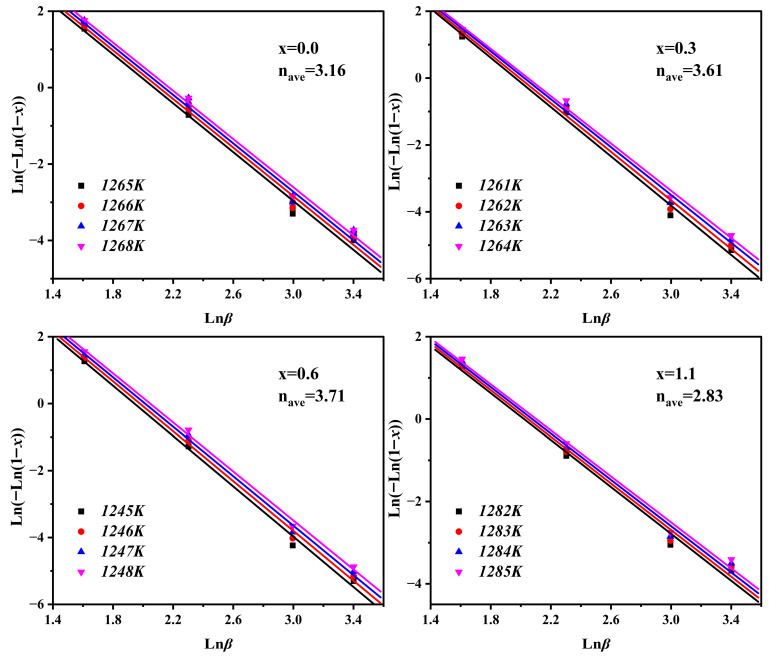
Plots of ln[−ln(1 − *x*)] versus ln*β* for parent glasses with different additions of Na_2_O.

As derived from Figure 5, the average Avrami exponent values (*n*_ave_) for samples with varying Na_2_O additions are 3.16, 3.61, 3.71, and 2.83, respectively. This indicates that the crystallization process of the parent glass is dominated by three-dimensional bulk crystal growth. Furthermore, the samples with x = 0.0, 0.3, and 0.6 exhibit *n*_ave_ > 3, suggesting that nucleation in these samples is interface-controlled. In contrast, the sample with x = 1.1 has *n*_ave_ < 3, indicating that its nucleation is diffusion-controlled [30,31,32]. As x increases from 0.0 to 0.6, nave increases from 3.16 to 3.71, implying that with the addition of Na_2_O, the nucleation behavior of the parent glass may gradually transition from a mechanism closer to site saturation toward continuous nucleation [36].

**Figure 6:**
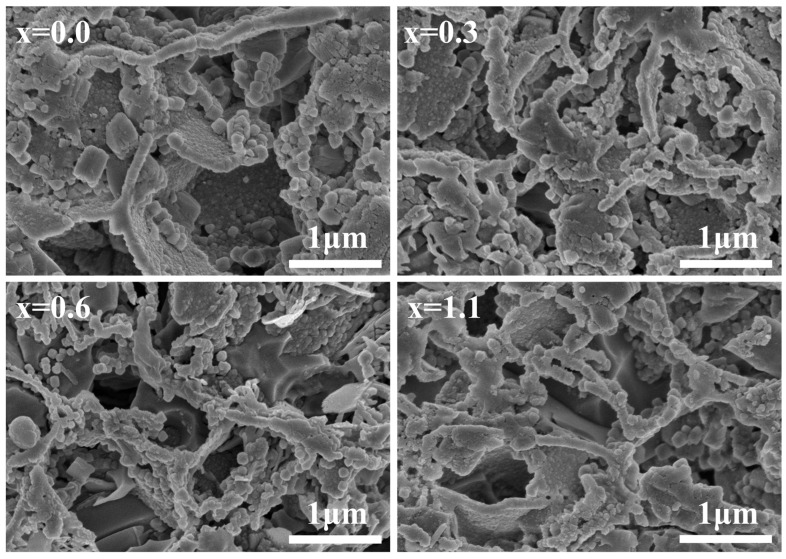
SEM micrographs of the etched surfaces of cordierite glass-ceramics with varying Na_2_O additions, prepared at their respective optimal sintering temperatures.

To verify the validity of the non-isothermal crystallization kinetics calculations, Figure 6 presents the SEM morphologies of acid-etched cordierite glass-ceramics prepared from parent glasses with varying Na_2_O additions at their respective optimal sintering temperatures. It can be observed that the crystal grains in all glass-ceramics are interconnected in space and exhibit a dense three-dimensional packing structure within the glass matrix. This is consistent with the three-dimensional crystal growth mechanism inferred from *n*.

### 3.3. Phase Evolution and Crystallization Behavior

**Figure 7:**
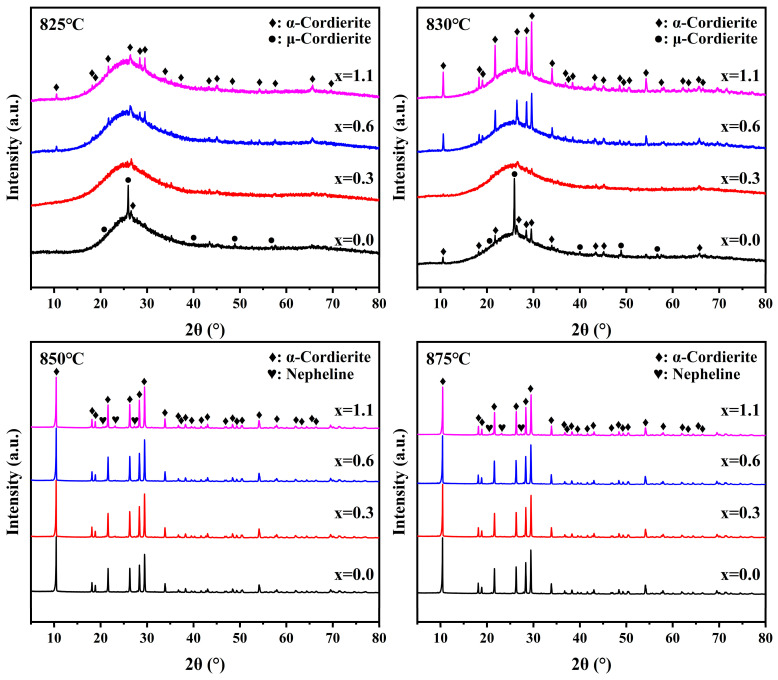
XRD patterns of parent glasses with different Na_2_O additions at different sintering temperature.

Figure 7 presents the XRD patterns of parent glasses with varying Na_2_O additions crystallized for 6 h at different heat treatment temperatures (825, 830, 850 and 875 °C). The glass-ceramic samples with x = 0 and x = 0.3 contain only two crystalline phases: μ-cordierite (PDF#97-002-4899) and α-cordierite (PDF#97-007-5634). However, for the samples with x = 0.6 and x = 1.1, a new crystalline phase, nepheline (NaAlSiO_4_: PDF#98-000-0327), appears. This indicates that the introduction of high concentrations of Na_2_O induces the formation of sodium-containing impurity phases. The crystallinity of glass-ceramic samples with varying Na_2_O additions, calculated via the peak-fitting method, is presented in Table 5.

At a sintering temperature of 825 °C, the blank sample exhibited a distinct amorphous hump, accompanied by weak diffraction peaks of μ-cordierite and trace peaks of α-cordierite, corresponding to a crystallinity of 4.92%. In contrast, the samples containing Na_2_O displayed a prominent amorphous hump along with weak α-cordierite diffraction peaks; notably, with increasing Na_2_O addition, the crystallinity of these samples rose from 1.80% to 3.18%. Upon elevating the sintering temperature to 830 °C, the diffraction intensities of both μ-cordierite and α-cordierite in the blank sample further intensified, with the crystallinity increasing to 8.88%. In contrast, the Na_2_O-modified samples exhibited enhanced α-cordierite diffraction intensity; notably, as the Na_2_O addition increased, the crystallinity of the samples rose from 2.40% to 16.69%. Combined with the results in Table 3, which indicate that the addition of Na_2_O facilitates early crystal precipitation, it is evident that the samples maintain a high α-cordierite content even in the absence of a substantial transformation from μ-cordierite to α-cordierite. The formation of α-cordierite is attributed to two distinct pathways: the phase transformation from μ-cordierite and the direct nucleation and growth of the α-phase [37]. Consequently, it can be inferred that Na_2_O suppresses the formation of the μ-cordierite phase and facilitates the direct crystallization of α-cordierite, thereby effectively shortening the overall crystallization sequence of the samples. This behavior may be attributed to the role of Na_2_O as a network modifier in the parent glass structure. The incorporation of Na_2_O is considered to depolymerize the glass network and increase the amount of non-bridging oxygen. According to the previous literature [22,23,24] and the structural evidence obtained in this work, such changes may be associated with reduced high-temperature viscosity and enhanced cation diffusion and local structural rearrangement during heat treatment. As a result, the direct reconstruction of structural units related to α-cordierite formation may be facilitated, making this crystallization pathway more favorable than the route involving transformation through the μ-cordierite intermediate phase. As evidenced by Table 5, the x = 0.3 and x = 0.6 samples attained their peak crystallinity values of 83.87% and 84.57% at 850 °C, respectively. In contrast, the x = 0.0 and x = 1.1 samples reached their maximum crystallinity (81.56% and 82.95%) at a higher temperature of 875 °C. This suggests that incorporating an appropriate amount of Na_2_O not only enhances the overall crystallinity but also shifts the crystallization process toward lower temperatures, effectively promoting premature crystal precipitation.

**Figure 8:**
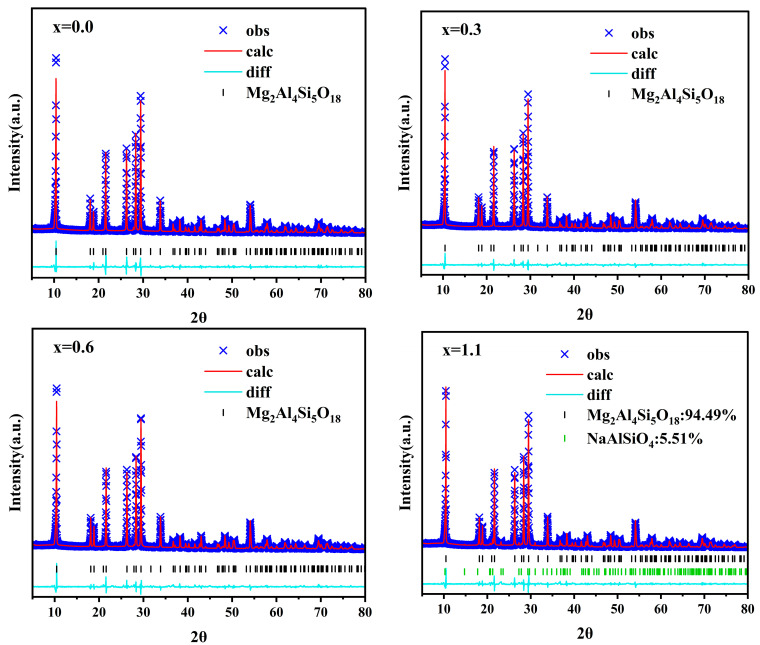
Rietveld refinement of parent glasses at their optimal sintering temperatures.

**Figure 9:**
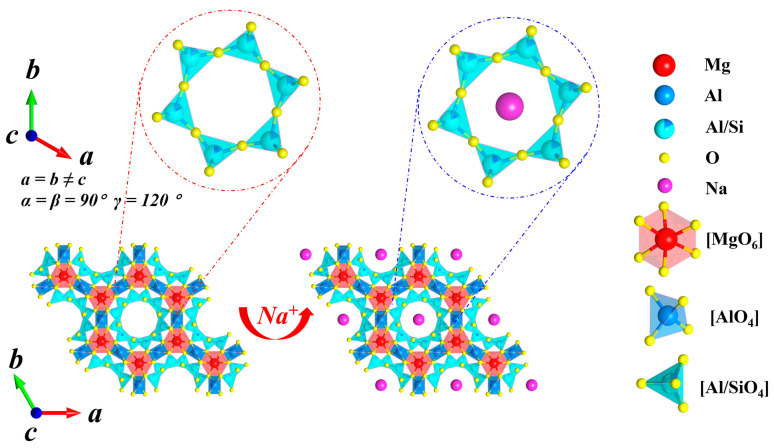
Schematic illustration of the structural distortion in the α-cordierite lattice induced by Na^+^ incorporation into the hexagonal ring channels.

Figure 8 displays the Rietveld refinement results for glasses with varying Na_2_O additions at their respective optimal sintering temperatures. Table 6 summarizes the lattice parameters obtained from the Rietveld refinement. The post-refinement reliability factors, *R_p_* and *R_wp_*, are both less than 10%, indicating the reliability of the refinement results. When x increases from 0.6 to 1.1, the proportion of the nepheline phase NaAlSiO_4_ rises from 0.99% to 5.51%. The presence of the nepheline phase hinders the grain boundary migration and grain growth of α-cordierite. This explains why the sample with x = 1.1 exhibits lower crystallinity at its optimal sintering temperature. Simultaneously, it can be observed that with the increase in Na_2_O addition, the unit cell volume of α-cordierite gradually expands. Overall, this trend manifests as an increase in the lattice parameters a and b, while c remains essentially unchanged. This result suggests a high probability that Na^+^ ions occupy the six-membered ring channels of α-cordierite. A schematic diagram illustrating this structural distortion is shown in Figure 9.

### 3.4. Structure of the Parent Glass

**Figure 10:**
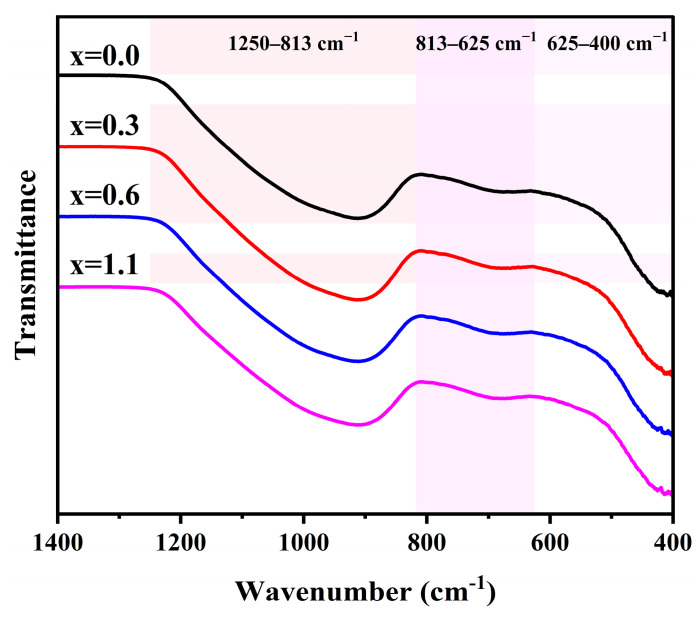
FTIR spectra of parent glasses with different additions of Na_2_O.

However, it should be emphasized that, due to the strong overlap among the fitted components, the Gaussian deconvolution inevitably involves a certain degree of uncertainty. Therefore, the FTIR peak-fitting results should be regarded as semi-quantitative evidence for structural evolution. To further verify the reliability of the analysis, XPS O 1s measurements were additionally performed on the parent glasses as an independent and complementary means of cross-validation. Figure 10 shows the FTIR spectra of parent glasses with varying Na_2_O additions. The FTIR spectra of all glass samples mainly consist of three regions: 1250–813 cm^−1^, 813–625 cm^−1^, and 625–400 cm^−1^. These bands can be attributed to the asymmetric stretching vibrations, symmetric stretching vibrations, and bending vibrations of the R-O-R bonds in [RO_4_] (R = Si or Al) tetrahedra, respectively [38].

**Figure 11:**
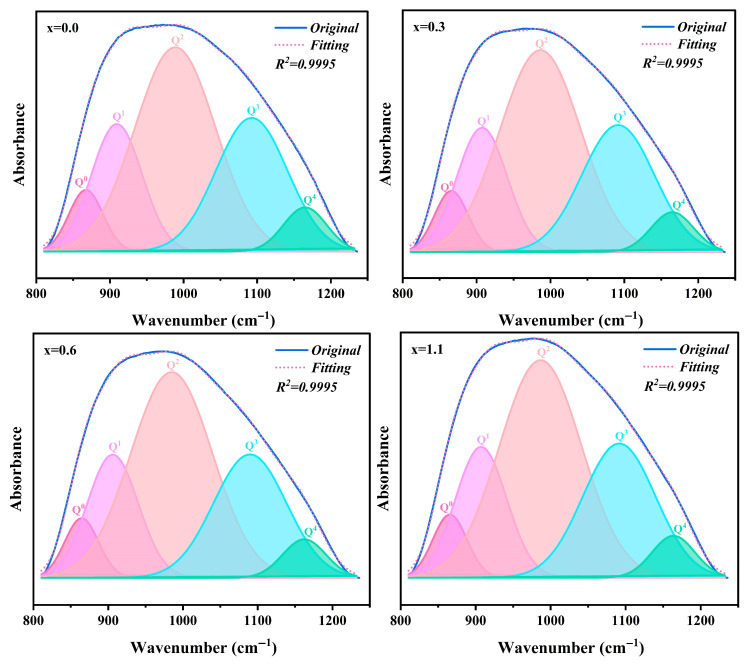
Deconvoluted FTIR spectra of parent glasses with different additions of Na_2_O.

As the Na_2_O addition increases from 0 to 1.1 wt.%, the absorption band in the 1250–813 cm^−1^ range undergoes a systematic shift toward lower wavenumbers. This phenomenon is primarily attributed to the depolymerization of the three-dimensional network structure of the parent glass induced by Na_2_O, which results in an increased concentration of NBOs. This specific absorption band originates from the superposition of vibrations from [SiO_4_] structural units with varying degrees of polymerization, denoted as Q^n^ species (n = 0–4) [39,40,41,42]. To semi-quantitatively evaluate the variations in the connectivity of the glass network, Gaussian deconvolution was employed to fit the spectral envelopes in this region, as illustrated in Figure 11. Table 7 summarizes the relative peak area percentages of the different Q^n^ species. With the increasing addition of Na_2_O, the relative contents of Q^3^ and Q^4^ decrease, whereas those of Q^0^, Q^1^ and Q^2^ exhibit an upward trend. This observation indicates that the incorporation of Na_2_O facilitates the transformation of highly polymerized structural units into less-connected ones, thereby depolymerizing the glass network. To quantify this trend, the ratio of NBO/BO, defined as (Q^0^ + Q^1^ + Q^2^)/(Q^3^ + Q^4^), was employed to characterize the relative evolution of the glass network connectivity. It can be observed that the NBO/BO ratio increases monotonically with higher Na_2_O concentrations.

**Figure 12:**
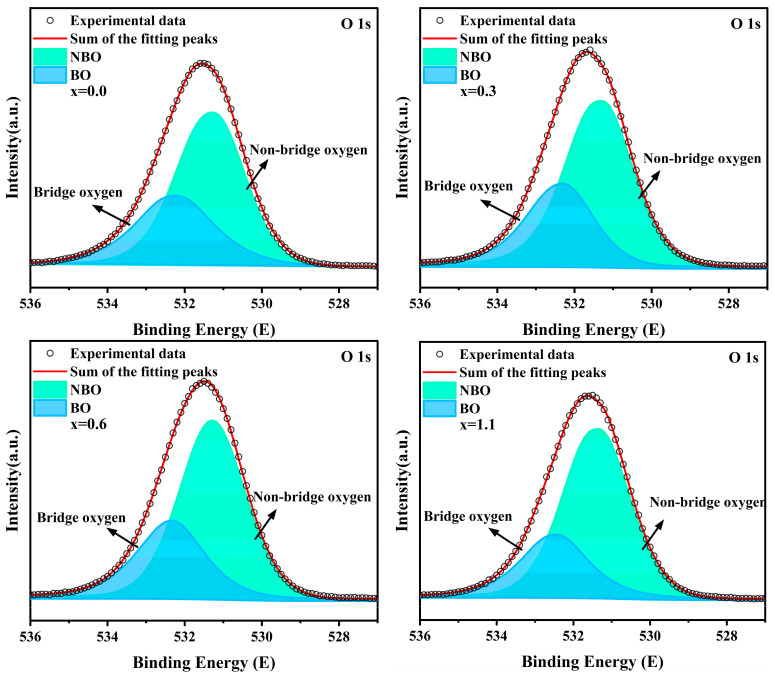
The detected and fitted O 1s spectra of glass samples.

Figure 12 displays the XPS O 1s spectra of parent glasses with varying Na_2_O additions, along with their corresponding peak fitting curves. The peak near 531.7 eV can be deconvoluted into two O 1s components: BO with a binding energy at approximately 532.2 eV and NBO with a binding energy at approximately 531.3 eV [43,44]. The detailed fitting results are presented in Table 8. As the Na_2_O addition increases, the NBO percentage in the parent glass rises from 64.74% to 71.46%, while the BO percentage decreases from 35.26% to 28.54%; correspondingly, the NBO/BO ratio increases from 1.84 to 2.50. Since FTIR primarily reflects bulk information while XPS focuses on surface information, there is a certain discrepancy in the NBO/BO values calculated by the two methods. However, the overall trends are consistent, which also aligns well with the variations in crystallization behavior exhibited in the DSC results.

### 3.5. Microstructure of Cordierite Glass-Ceramics

**Figure 13:**
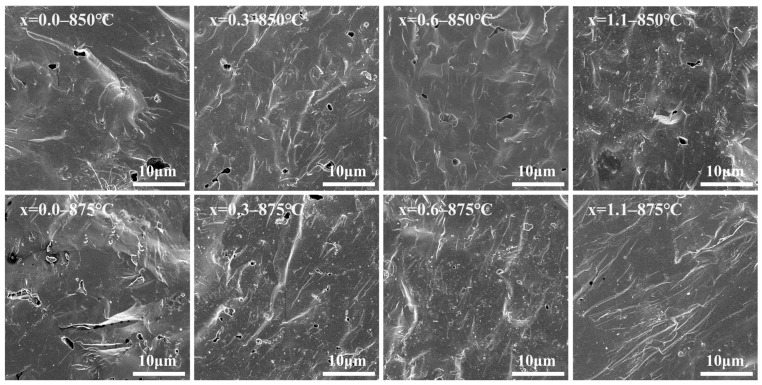
SEM images of fractured surfaces for glass-ceramics prepared with Na_2_O additions at 850 °C and 875 °C.

Figure 13 presents the fracture surface morphologies of the glass-ceramic samples with varying Na_2_O additions sintered at 850 °C and 875 °C. For glass-ceramics fabricated through the sintering route, the densification process is primarily governed by the viscous flow mass transport mechanism, which involves distinct stages including particle rearrangement, neck growth, and the subsequent shrinkage and closure of pores [45,46,47]. At a sintering temperature of 850 °C, the blank sample exhibited numerous irregular pores, with some localized pores being relatively large. As the Na_2_O addition increased to 0.6 wt.%, both the quantity and size of the pores progressively decreased. This trend is attributed to the fact that an optimal addition of Na_2_O can effectively reduce glass viscosity by decreasing the degree of network polymerization and broadening the densification sintering window. Consequently, particle rearrangement and pore closure driven by viscous flow are significantly enhanced, facilitating densification at lower temperatures. However, as the Na_2_O addition increased to 1.1 wt.%, an anomalous resurgence in the number of pores was observed. However, when the Na_2_O addition was increased to 1.1 wt.%, the number of pores in the samples anomalously increased. This was primarily attributed to the premature precipitation of crystals induced by excessive Na_2_O, which hindered the sintering densification of the glass-ceramics (as evidenced by the XRD patterns, the sample with x = 1.1 exhibited the highest crystallinity at the initial stage of sintering). During the sintering of glass-ceramics, the continuous precipitation of crystals and the subsequent grain growth restrict the flow paths of the residual glass phase, thereby increasing the effective viscosity of the system. This phenomenon inherently narrows the densification sintering window, rendering the closure of residual pores increasingly difficult or even triggering pore coarsening [48]. As the sintering temperature was increased to 875 °C, the porosity of the samples increased to a certain extent. In addition to the inhibitory effects of continuous crystallization and grain growth on densification, this phenomenon may also be attributed to the excessively high sintering temperature, which causes the base glass to soften too rapidly, leading to the premature closure of gas escape channels and the entrapment of part of the gas within the glass-ceramic, where it forms closed pores [49]. At a sintering temperature of 850 °C and a Na_2_O addition of 0.6 wt.%, the samples exhibit the optimal degree of densification, characterized by minimal porosity and a highly uniform pore size distribution.

### 3.6. Density

**Figure 14:**
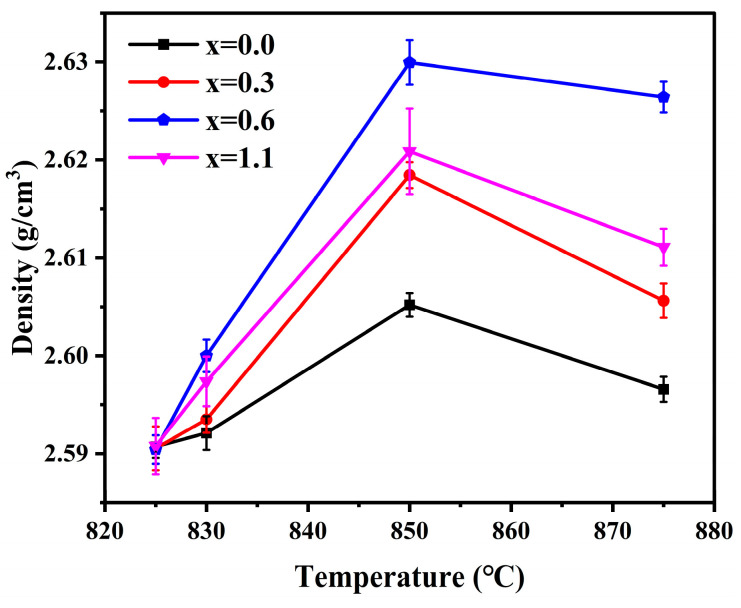
Density of glass-ceramics prepared with varying Na_2_O additions at different sintering temperature.

Figure 14 illustrates the densities of the glass-ceramic samples with varying Na_2_O additions prepared at different sintering temperatures. Overall, the densities of all samples exhibited a consistent upward trend as the sintering temperature was elevated from 825 °C to 850 °C. This is attributed to the synergistic enhancement of both crystallization and densification facilitated by the increasing sintering temperature. On one hand, the elevated temperature effectively lowers the high-temperature viscosity of the glass phase, which promotes mass transport via viscous flow and accelerates pore closure. On the other hand, cordierite crystals possess a more compact structural packing compared to the amorphous glass phase; consequently, the increase in crystalline content significantly contributes to the improvement of the overall density. Within this sintering interval, the variation in sample density as a function of Na_2_O addition is likewise influenced by the two aforementioned factors. As previously noted, an appropriate amount of Na_2_O can increase the overall density of the samples by promoting densification and enhancing their crystallinity. However, as evidenced by Table 5 and Figure 14, excessive Na_2_O triggered premature crystal precipitation, which hindered the sintering densification and crystallization of the samples, thereby leading to a decline in density. It is noteworthy that when the sintering temperature was further increased to 875 °C, the density of the glass-ceramics exhibited a certain degree of decline. This phenomenon could be attributed to the excessively low viscosity of the parent glass at higher sintering temperatures, which led to premature surface sealing and hindered the escape of internal gases, ultimately resulting in the formation of closed pores. At a sintering temperature of 850 °C and an Na_2_O addition of 0.6 wt.%, the samples exhibited the highest degree of crystallinity and the most pronounced densification, characterized by minimal pore quantity and size. Consequently, a peak density of 2.63 g/cm^3^ was achieved.

### 3.7. Flexural Strength

**Figure 15:**
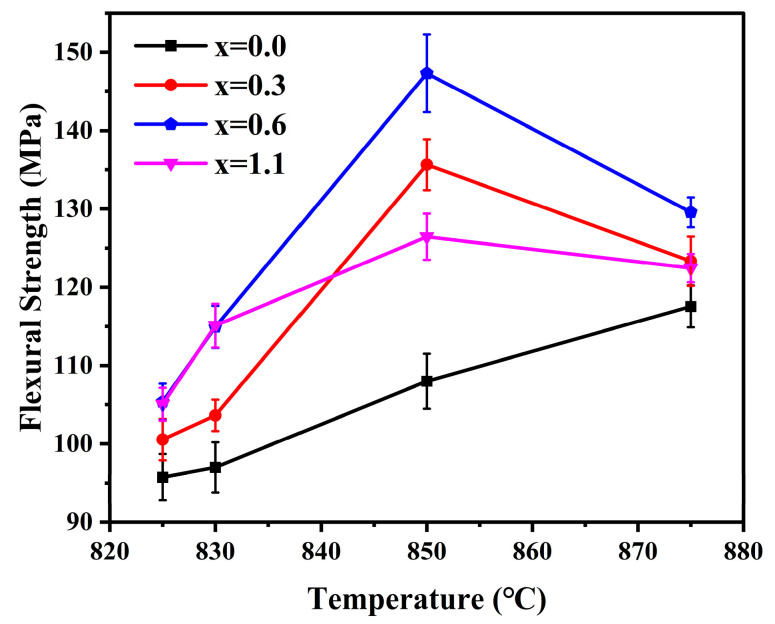
Flexural strength of glass-ceramics prepared with varying Na_2_O additions at different sintering temperature.

Figure 15 illustrates the flexural strength of the glass-ceramics prepared with varying Na_2_O additions at different sintering temperatures. Overall, the variation trend of the flexural strength is highly consistent with that of the density, both being governed by the synergistic effect of the crystalline phase content and the degree of densification. The flexural strength of the samples exhibited a monotonic increase as the sintering temperature was raised from 825 °C to 850 °C. This phenomenon can be ascribed to two primary factors. First, the elevated sintering temperature promoted the precipitation and growth of cordierite crystals. Since cordierite crystals possess a higher elastic modulus and fracture surface energy than the amorphous glass phase, the increased crystallinity effectively enhanced the crack propagation resistance of the glass-ceramics [50,51]. Second, the higher sintering temperature facilitated the elimination of pores, significantly reducing the quantity and size of stress concentration sites, thereby improving the flexural strength of the samples [52]. As the sintering temperature was further increased to 875 °C, the flexural strength of the glass-ceramics exhibited a certain degree of decline. In conjunction with the density analysis, this can be attributed to the excessively low viscosity of the parent glass at higher sintering temperatures, which led to premature surface sealing. This process prevented the escape of internal gases and resulted in the formation of closed pores, thereby increasing the quantity and size of stress concentration sites and ultimately reducing the flexural strength of the samples. Notably, despite the presence of a greater number of internal pores, the crystallinity of the blank sample increased from 77.76% to 83.56%. The sustained enhancement in flexural strength may be attributed to the fact that the positive contribution from the increased crystalline phase content outweighed the detrimental impact of the increased porosity.

In addition to the synergistic influence of the crystalline phase content and the degree of densification, the evolution of the samples’ flexural strength with varying Na_2_O addition was also governed by factors such as lattice distortion, microcracks, and regions of phase boundary mismatch. The structural distortion of the six-membered rings, induced by the incorporation of Na^+^ ions into the α-cordierite channels, may also alleviate the internal stress of the glass-ceramics by hindering the rotation of the rings, thereby enhancing the overall flexural strength of the samples [53]. Concurrently, the addition of Na_2_O suppressed the transformation from metastable μ-cordierite to α-cordierite, effectively mitigating the risk of microcracking associated with the volume changes during phase transition [51]. Notably, as evidenced by Table 5, the sample with x = 1.1 contains a certain amount of the NaAlSiO_4_ phase. The lattice and thermal expansion mismatch between the NaAlSiO_4_ secondary phase and the primary α-cordierite phase tends to introduce residual stresses at the phase boundary regions. These regions preferentially act as crack initiation sites under external loading, thereby leading to a reduction in the flexural strength of the samples [54]. Ultimately, a maximum flexural strength of 147.3 Mpa was achieved at a sintering temperature of 850 °C with a Na_2_O addition of 0.6 wt.%.

### 3.8. Vickers Hardness

**Figure 16:**
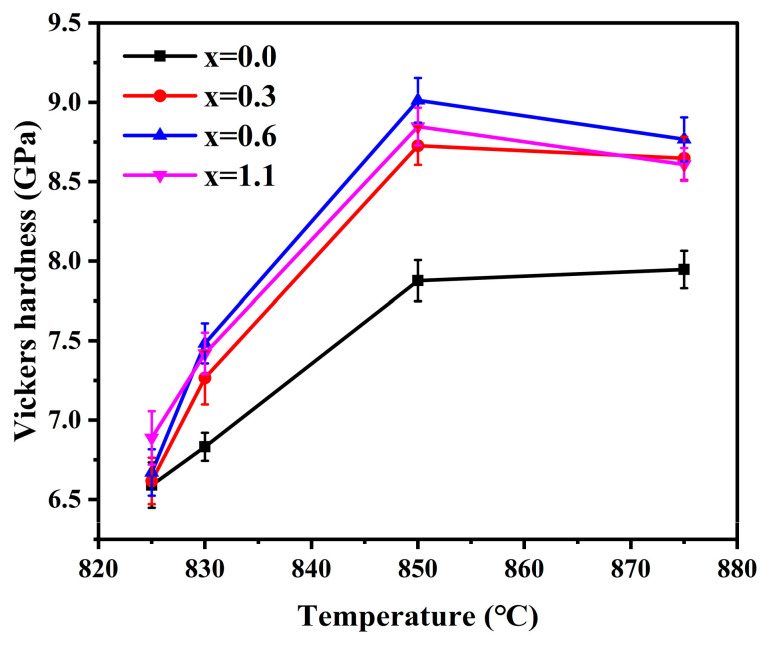
Vickers hardness of glass-ceramics prepared with varying Na_2_O additions at different sintering temperature.

Figure 16 presents the Vickers hardness of glass-ceramics with different Na_2_O addition amounts prepared at different sintering temperatures. Overall, the Vickers hardness of the samples first increased and then decreased with increasing sintering temperature, and after the addition of Na_2_O, the overall hardness of the samples was higher than that of the sample without Na_2_O addition.

The Vickers hardness of glass-ceramics is mainly governed by the combined effects of microstructure, degree of densification, residual glassy phase, and the type and amounts of crystalline phases. As the sintering temperature increased from 825 °C to 850 °C, the Vickers hardness of all samples gradually increased. This indicates that, within this temperature range, the increase in sintering temperature is beneficial to improving the densification of the samples, reducing pore defects, and promoting the crystallization of α-cordierite, thereby jointly enhancing the hardness. Compared with the residual glassy phase, the α-cordierite crystalline phase generally possesses higher local rigidity [50,51], whereas internal defects such as pores reduce the effective load-bearing area of the material and induce stress concentration under the indentation stress field, thereby weakening the resistance of the material to indentation-induced deformation [52]. When the sintering temperature was further increased to 875 °C, the Vickers hardness of the samples decreased to some extent, which may be associated with factors such as the excessively rapid viscous flow of the glass melt at higher sintering temperatures, leading to the formation of local closed pores and grain coarsening.

The introduction of an appropriate Na_2_O addition amount is beneficial for improving the Vickers hardness of the samples. This is mainly associated with the role of Na_2_O in promoting low-temperature densification, enhancing the crystallization of α-cordierite, and reducing pore defects. It is worth noting that, as shown in Table 5, when the sintering temperature reached 875 °C, the Vickers hardness of the x = 1.1 sample decreased to a certain extent. In addition to the reasons discussed above, this may also be related to the presence of a certain amount of the secondary NaAlSiO_4_ crystalline phase in the sample. A certain degree of lattice and thermal expansion mismatch may exist between the NaAlSiO_4_ phase and the primary α-cordierite crystalline phase, thereby introducing residual stress in the interfacial regions and consequently reducing the Vickers hardness [54]. When the sintering temperature was 850 °C and the Na_2_O addition amount was 0.6 wt.%, the sample exhibited the highest crystallinity, the smallest number and size of pores, and the greatest degree of densification, with the Vickers hardness reaching a maximum value of 9.01 Gpa.

### 3.9. Dielectric Properties

**Figure 17:**
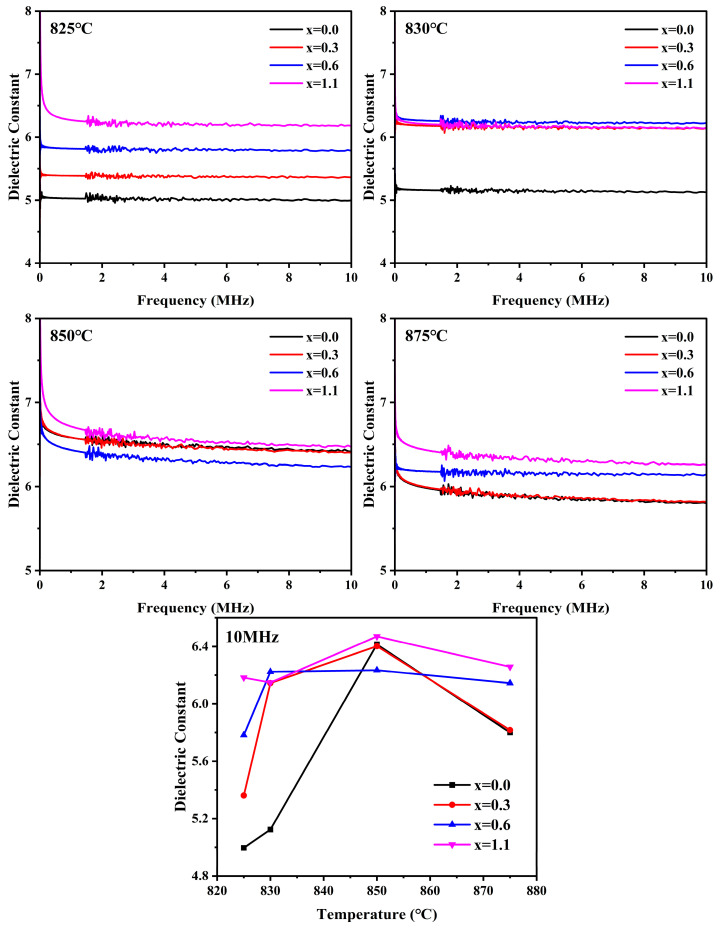
Dielectric constant of glass-ceramics prepared with varying Na_2_O additions at different sintering temperature versus frequency in the range 20 Hz–10 MHz and versus temperature at 10 MHz.

Figure 17 illustrates the frequency dependence (20 Hz–10 MHz) of the dielectric constant for cordierite glass-ceramics with varying Na_2_O additions prepared at different sintering temperatures, as well as the dielectric constant values at 10 MHz. As shown in Figure 16 the dielectric constant of the samples decreases gradually with increasing frequency. This phenomenon is attributed to the fact that at low frequencies, various internal polarization mechanisms can sufficiently respond to the alternating electric field. However, as the frequency increases, the oscillation period of the electric field progressively shortens until it is less than the relaxation time required for the internal polarization response. Consequently, certain polarization mechanisms, such as interfacial and dipolar polarization, fail to keep pace with the rapid oscillations of the electric field, leading to the phenomenon of polarization lag [55,56].

The dielectric constant of glass-ceramics is governed by a combination of factors, including the type and content of crystalline phases, the composition of the residual glassy matrix, the microstructure, and interface effects. Given that the dielectric constant of α-cordierite (~5) is lower than that of the residual glass phase, an increase in its volume fraction will, to a certain extent, lower the effective dielectric constant of the samples [5]. Furthermore, the presence of pores is analogous to the introduction of a low-permittivity air phase (~1), which significantly reduces the overall dielectric constant of the glass-ceramics [57]. In this study, the crystalline phase and its content, the glass phase composition, and the degree of densification are identified as the primary determinants of the dielectric constant.

At sintering temperatures of 825 °C and 830 °C, the glass-ceramic samples were in the initial sintering stage, consisting of a predominant glassy matrix and a minor crystalline fraction. The low degree of densification and high porosity at this stage resulted in a relatively low overall dielectric constant. At this stage, the dielectric constant of the glass-ceramics is predominantly governed by the composition of the glassy matrix and the presence of porosity. With increasing Na_2_O addition, the dielectric constant of the samples exhibits a gradual upward trend. This is primarily attributed to the role of Na_2_O as a network modifier; its incorporation disrupts the three-dimensional glass network, generating a higher concentration of NBOs. The resulting decrease in the degree of network polymerization enhances the contribution of ionic polarization [58]. Concurrently, the addition of Na_2_O promotes sintering densification, thereby mitigating the ‘dilution effect’ that pores exert on the overall permittivity of the glass-ceramics. As the sintering temperature was increased to 850 °C, both the crystallinity and densification of the samples exhibited a significant increase, as evidenced by Table 5 and Figure 16. At this stage, the evolution of the overall dielectric constant was primarily governed by a competitive mechanism between the degree of densification (positive contribution) and crystallization (negative contribution). Although the precipitation of the primary α-cordierite phase tends to decrease the overall dielectric constant, the enhancement in densification triggered by the elevated sintering temperature remains the predominant factor, ultimately resulting in a net increase in the dielectric constant of the samples. Notably, as the Na_2_O addition increased, the dielectric constant of the samples exhibited an initial decrease followed by a subsequent increase, which is consistent with the variation trend of crystallinity. This phenomenon can be attributed to the high degree of densification achieved at 850 °C; under these conditions, the volume fraction of the crystalline phase becomes the dominant factor governing the dielectric properties. Regarding the x = 1.1 sample, although its crystallinity is higher than that of the blank sample, the presence of 4.26% of the high-permittivity NaAlSiO_4_ phase results in a dielectric constant that exceeds that of the blank sample. As the sintering temperature was further raised to 875 °C, the crystallinity of the samples showed no significant variation; however, the degree of densification declined to some extent, accompanied by an increase in porosity, which ultimately led to a reduction in the dielectric constant.

**Figure 18:**
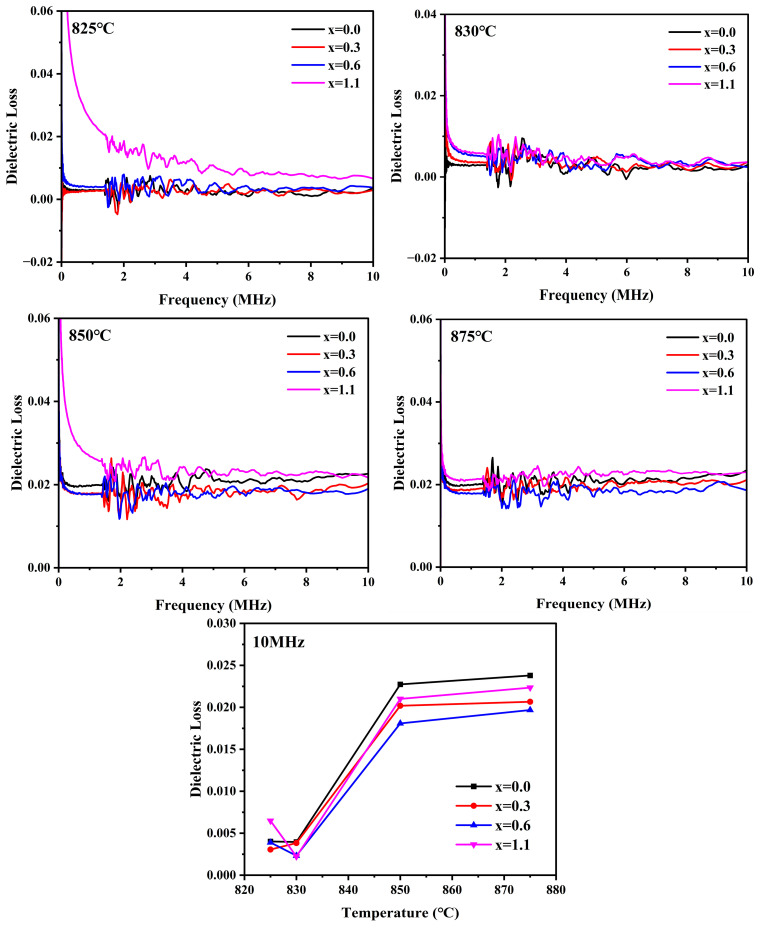
Dielectric loss of glass-ceramics prepared with varying Na_2_O additions at different sintering temperature versus frequency in the range 20 Hz–10 MHz and versus temperature at 10 MHz.

Figure 18 illustrates the frequency dependence (20 Hz–10 MHz) of the dielectric loss for cordierite glass-ceramics prepared from parent glasses with varying Na_2_O additions at different sintering temperatures, as well as the dielectric loss values at 10 MHz. It can be observed that the variation trend of the dielectric loss exhibits distinct staged characteristics, which are closely associated with the phase transition kinetics and microstructural evolution of the samples. At sintering temperatures of 825 °C and 830 °C, the samples were in the initial sintering stage. During this phase, the dielectric loss was predominantly governed by the conduction loss of the residual glassy matrix and the Maxwell–Wagner interfacial polarization loss at the gas–solid interfaces [59]. The addition of Na_2_O acts to disrupt the glass network structure, leading to an increase in NBO content and a corresponding decrease in BO content. This reduction in the degree of network polymerization facilitates the migration of charge carriers, thereby intensifying the conduction loss and ultimately elevating the overall dielectric loss of the samples [60]. Simultaneously, Na_2_O lowers the viscosity of the glass melt and promotes sintering densification, which effectively inhibits pore-induced interfacial polarization effects, thus contributing to a reduction in the overall dielectric loss. At 825 °C, the dielectric loss of the sample with x = 1.1 was significantly higher than that of the other compositions, as the conduction loss induced by the high Na^+^ concentration became the dominant factor. Conversely, at 830 °C, the sample with x = 0.6 exhibited the lowest dielectric loss, attributed to its optimal densification and minimal pore defects, which effectively suppressed interfacial polarization. The evolution of the dielectric loss with varying Na_2_O addition is fundamentally the result of a competitive interplay between the suppression of interfacial polarization through densification and the enhancement of conduction loss induced by Na^+^ ions. As the sintering temperature was further increased to 850 °C and 875 °C, the dielectric loss of all samples exhibited a pronounced, step-like increase, which was accompanied by the extensive precipitation of the primary α-cordierite phase. This phenomenon is the result of the coupling of multiple effects. Both elevated sintering temperatures and appropriate additions of Na_2_O effectively enhance the crystallinity of the samples. Given the low intrinsic dielectric loss of α-cordierite crystals, an increase in crystallinity should theoretically reduce the overall dielectric loss. However, this increase in crystallinity concurrently leads to a deterioration in dielectric properties. Firstly, as continuous crystallization proceeds, the added Na^+^ ions and impurity ions (such as Na^+^, K^+^ and Ca^2+^) originating from the perlite tailings are excluded from the cordierite crystal lattice and consequently become highly concentrated within the diminishing residual glass phase. This enrichment significantly elevates the concentration of polarizing ions in the residual glass, thereby inducing intense dipolar relaxation loss [61]. Secondly, the rapid increase in crystallinity generates a vast number of crystal-glass interfaces. Charges tend to accumulate at these interfaces, significantly potentiating the interfacial polarization effect [62,63]. Notably, as illustrated in Figure 5, excessive Na_2_O addition induces the formation of the NaAlSiO_4_ phase. This secondary phase possesses a high intrinsic dielectric loss, which further aggravates the overall loss of the system. At 850 °C, the dielectric loss was predominantly governed by the enrichment of the residual phase and interfacial polarization effects. Specifically, the x = 0.6 sample exhibited the minimum dielectric loss, owing to its highest bulk density and the fewest structural defects. Conversely, the x = 0.0 sample showed the highest loss due to its poor degree of densification and the significant presence of pore defects. As the sintering temperature was further elevated to 875 °C, the degree of densification of the samples declined; however, the dielectric loss remained at a sustained high level. In summary, the dielectric loss of the samples at this stage is predominantly governed by a competitive interplay between the reduction in intrinsic loss driven by the enhancement of crystallinity and the elevation in loss resulting from residual phase enrichment and intensified interfacial polarization. Na_2_O regulates this competitive relationship and the final dielectric loss through the modulation of the glass network structure, densification kinetics, crystallization behavior, and the formation of secondary phases.

### 3.10. Thermal Expansion Properties

**Figure 19:**
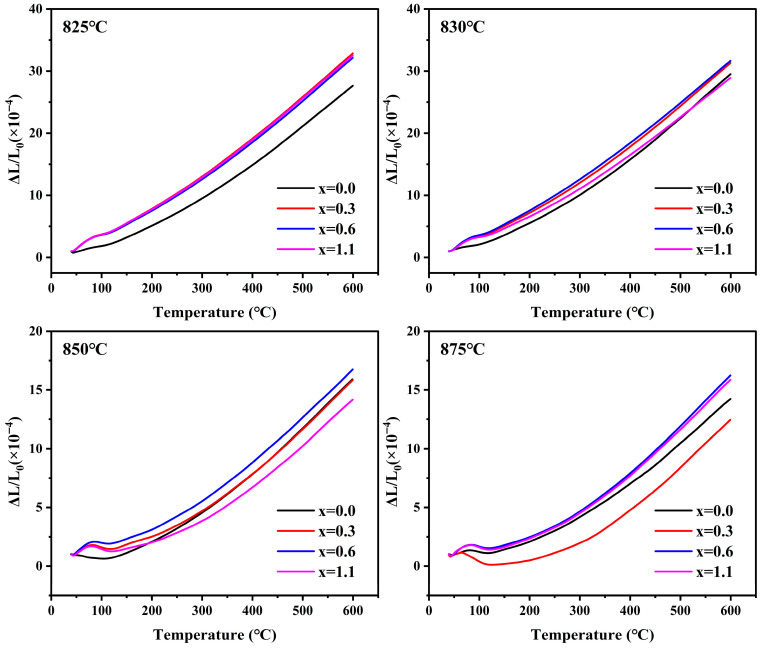
Thermal expansion behavior of the glass-ceramics prepared at different sintering temperatures.

Figure 19 illustrates the thermal expansion behavior in the range of 40 °C to 600 °C for cordierite glass-ceramics prepared at different sintering temperatures. The specific values of the average CTE calculated in the 100–600 °C interval are listed in Table 9. When the sintering temperature increases from 825 °C to 850 °C, the average CTE of the samples decreased significantly. This change is primarily related to the evolution of the crystalline phases. As the sintering temperature rose, the low-thermal-expansion α-cordierite phase gradually formed, and its content increased, while the relative content of the high-thermal-expansion residual glass phase decreased. As a result, the system transitioned from being glass-phase-dominated to crystal-phase-dominated, leading to a significant reduction in the overall average CTE of the samples. When the sintering temperature was further raised to 875 °C, the average CTE of the x = 0.0–0.6 samples continued to decrease, indicating that, in addition to changes in crystalline phase composition, alterations in the microstructure of the samples might also influence their thermal expansion response. As shown in Figure 14, the density of the samples decreased to some extent, suggesting that the densification of the samples was reduced, and the internal porosity might have increased. Such microstructural changes could weaken the apparent thermal strain response of the glass-ceramics, resulting in a lower measured average CTE [64,65]. Furthermore, the thermal expansion anisotropy of the cordierite crystal axes may induce microcracks at the domain boundaries, which could also contribute to the reduction in the overall CTE of the samples [66]. For the x = 1.1 sample, its average CTE increased anomalously. This is likely related to the increased relative proportion of NaAlSiO_4_ phase in the sample. Since the CTE of NaAlSiO_4_ is higher than that of α-cordierite, the increase in its proportion would raise the overall average CTE of the sample [67]. Furthermore, by comparing the average CTEs of glass-ceramic samples with varying Na_2_O additions prepared at their respective optimal sintering temperatures, with increasing Na_2_O additions, the crystallinity of the samples first increased and then decreased, whereas the average CTE exhibited an increasing trend. Predecki et al. [68] proposed that the thermal expansion driving force of cordierite crystals is the thermal deformation of [MgO_6_] octahedra. Due to the weak bond strength between Mg and O, this results in expansion along the a-axis and contraction along the c-axis, manifesting as anisotropic thermal effects that cause the flattening of the [MgO_6_] octahedra. Therefore, when Na^+^ enter the six-membered ring channels of α-cordierite, they may hinder the thermal contraction along the c-axis of α-cordierite. This reduces the contribution of anisotropic contraction, thereby increasing the average CTE of the sample.

## 4. Conclusions

In this study, low-temperature densification and comprehensive property optimization of perlite tailings-based α-cordierite glass-ceramics were achieved through the synergistic regulation of Na_2_O addition amount and sintering temperature. The results indicate that Na_2_O, acting as a network modifier, promotes the transformation of highly polymerized structural units into less polymerized ones in the base glass, thereby reducing the degree of polymerization of the glass network and facilitating structural rearrangement, viscous flow, and crystallization at lower temperatures. On this basis, Na_2_O further affects the crystallization pathway and kinetic behavior of cordierite. The depolymerization of the base glass network helps reduce the crystallization activation energy of α-cordierite; meanwhile, the introduction of Na_2_O weakens the dependence of the system on the metastable μ-cordierite intermediate pathway, suppresses the tendency toward crystallization via transformation through the μ-cordierite intermediate phase, and promotes the direct precipitation of the primary α-cordierite crystalline phase. Non-isothermal crystallization kinetic analysis shows that the crystallization process of all samples is dominated by three-dimensional crystal growth controlled by bulk nucleation, and that with increasing Na_2_O addition amount, the nucleation behavior gradually shifts from being closer to site saturation toward continuous nucleation. The Na_2_O addition amount and sintering temperature also jointly influence the mechanical properties, dielectric properties, and thermal expansion behavior of the materials by regulating the degree of densification, the composition and relative contents of crystalline phases and residual glassy phase, as well as the microstructure. An appropriate Na_2_O addition amount is beneficial to low-temperature densification and the precipitation of α-cordierite, thereby improving the overall properties; however, excessive Na_2_O induces the formation of the secondary NaAlSiO_4_ crystalline phase, alters the phase composition of the system, and deteriorates microstructural uniformity, which is unfavorable for further property enhancement. When the Na_2_O addition amount was 0.6 wt.% and the sintering temperature was 850 °C, the sample exhibited the optimal overall performance: εr = 5.82 and tanδ = 1.80 × 10^−2^ at 10 MHz, a high flexural strength of 147.3 MPa, a Vickers hardness of 9.01 GPa, and a suitable CTE of 2.96 × 10^−6^ K^−1^ which is close to that of Si. This study provides a new approach for the high-value utilization of perlite tailings and also offers a certain theoretical reference for the design of low-cost LTCC substrate materials.

## Figures and Tables

**Table 1:** Chemical compositions of perlite tailings (wt.%).

Compositions	SiO_2_	Al_2_O_3_	K_2_O	Na_2_O	CaO	Fe_2_O_3_	MgO	TiO_2_	Others	LOI
Content	70.92	11.73	4.73	2.76	1.15	1.02	0.42	0.10	1.75	5.42

**Table 2:** Chemical compositions of parent glasses (wt.%).

Sample	Added Na_2_O, x	MgO	Al_2_O_3_	SiO_2_	Total Na_2_O	Other
x = 0.0	0.00	16.34	31.79	46.83	1.24	3.80
x = 0.3	0.30	16.29	31.69	46.68	1.54	3.80
x = 0.6	0.60	16.23	31.59	46.54	1.84	3.80
x = 1.1	1.10	16.15	31.42	46.29	2.34	3.80

**Table 3:** Thermal parameters of parent glasses obtained from DSC.

Samples	*T*_g_ (°C)	*T*_o_ (°C)	*T*_p1_ (°C)	*T*_p2_ (°C)
x = 0.0	806.6	948.8	970.1	1031.6
x = 0.3	801.5	944.3	969.0	1030.5
x = 0.6	798.3	932.7	953.7	1010.4
x = 1.1	796.0	955.0	986.2	1056.0

**Table 4:** The crystallization activation energy *E* calculated by the Kissinger method and Owaza method.

Sample	Kissinger Method *E*/(kJ/mol)	Ozawa Method *E*/(kJ/mol)
x = 0.0	411.66	432.86
x = 0.3	403.36	424.55
x = 0.6	437.08	458.00
x = 1.1	326.81	348.31

**Table 5:** Degree of crystallinity for the glass-ceramic specimens with varying Na_2_O additions.

Sample	Crystallinity (%)			
	825 °C	830 °C	850 °C	875 °C
x = 0.0	4.92 (±0.04)	8.88 (±0.14)	37.51 (±0.44)	81.56 (±5.12)
	3.96 ***α*** + 0.96 μ	6.00 α + 2.88 μ	36.96 α + 0.55 μ	81.56 α
x = 0.3	1.80 (±0.06)	2.40 (±0.08)	83.87 (±0.97)	80.95 (±3.50)
	1.80 α	2.40 α	83.87 α	80.95 α
x = 0.6	2.72 (±0.50)	12.6 (±0.15)	84.57 (±1.32)	83.00 (±5.85)
	2.72 α	12.6 α	84.57 α	83.00 α
x = 1.1	3.18 (±0.50)	16.69 (±1.01)	81.36 (±1.12)	82.95 (±4.54)
	3.18 α	16.69 α	77.10 α + 4.26 NaAlSiO_4_	78.38 α + 4.57 NaAlSiO_4_

**Table 6:** Refined lattice parameters for the glass-ceramic specimens with varying Na_2_O additions.

Structural Parameter	Sample			
	x = 0.0	x = 0.3	x = 0.6	x = 1.1
*a* (Å)	9.7712	9.7737	9.7753	9.7756
*b* (Å)	9.7712	9.7737	9.7753	9.7756
*c* (Å)	9.3977	9.3970	9.3966	9.3968
*V* (Å^3^)	777.358	777.393	777.601	777.648
*R_p_* (%)	6.63	5.61	6.57	6.78
*R_wp_* (%)	8.72	7.50	8.59	8.83
*χ* ^2^	2.97	2.47	2.90	2.91

**Table 7:** Fitting data of parent glasses with different additions of Na_2_O.

Sample	*Q* ^0^	*Q* ^1^	*Q* ^2^	*Q* ^3^	*Q* ^4^	NBO/BO
x = 0.0	5.771	17.681	45.140	26.200	5.209	2.184
x = 0.3	5.782	17.611	45.891	25.952	4.766	2.255
x = 0.6	5.651	17.463	46.655	25.520	4.7111	2.308
x = 1.1	5.644	17.555	46.930	25.214	4.657	2.348

**Table 8:** Binding energies, relative percentage of NBO and BO, and NBO/BO ratio for glass samples.

Sample	Non-Bridging Oxygen		Bridging Oxygen		NBO/BO
	Peak Position/eV	Percentage/%	Peak Position/eV	Percentage/%	
x = 0.0	531.26	64.74	532.21	35.26	1.84
x = 0.3	531.28	67.09	532.30	32.91	2.04
x = 0.6	531.29	68.26	532.34	31.74	2.15
x = 1.1	531.33	71.46	532.42	28.54	2.50

**Table 9:** Coefficients of average thermal expansion (100–600 °C) of glass-ceramics sintered from parent glasses including different Na_2_O additions at different sintering temperature.

Sample	CTE (×10^−6^ K^−1^)			
	825 °C	830 °C	850 °C	875 °C
x = 0.0	5.7136	5.4883	3.0607	2.6076
x = 0.3	5.8316	5.5686	2.6525	2.4183
x = 0.6	5.7144	5.6177	2.9564	2.9181
x = 1.1	5.7726	5.1339	2.5489	2.9581

## Data Availability

The original contributions presented in this study are included in the article. Further inquiries can be directed to the corresponding authors.
